# Study on Qualitative Impact Damage of Loquats Using Hyperspectral Technology Coupled with Texture Features

**DOI:** 10.3390/foods11162444

**Published:** 2022-08-13

**Authors:** Bin Li, Zhaoyang Han, Qiu Wang, Zhaoxiang Sun, Yande Liu

**Affiliations:** National and Local Joint Engineering Research Center of Fruit Intelligent Photoelectric Detection Technology and Equipment, School of Mechatronics & Vehicle Engineering, East China Jiaotong University, Nanchang 330013, China

**Keywords:** loquats, band radio image, gray level co-occurrence matrix, multispectral analysis method, morphological segmentation method

## Abstract

Bruising is one of the main problems in the post-harvest grading and processing of ‘*Zaozhong 6*’ loquats, reducing the economic value of loquats, and even food quality and safety problems are caused by it. Therefore, one of the main tasks in the post-harvest processing of loquats is to detect whether loquats are bruised, as well as the degree of bruising of loquats, to reduce the loss by proper treatment. An appropriate dimensionality reduction method can be used to reduce the redundancy of variables and improve the detection speed. The multispectral analysis method (MAM) has the advantage of accurate, rapid, and nondestructive detection, which was proposed to identify the different bruising degrees of loquats in this study. Firstly, the visible and near-infrared region (Vis–NIR, 400–1000 nm), the visible region (Vis, 400–780 nm), and the near-infrared region (NIR, 781–1000 nm) were analyzed using principal component analysis (PCA) to obtain the spectral regions and PC vectors, which could be used to effectively distinguish bruised loquats from normal loquats. Then, based on the selected second PC (PC2) score images, a morphological segmentation method (MSM) was proposed to distinguish bruised loquats from normal loquats. Furthermore, the weight coefficients of corresponding wavelength points of different degrees of bruising of loquats were analyzed, and the local extreme points and both sides of the interval were selected as the characteristic wavelength points for multi-spectral image processing. A gray level co-occurrence matrix (GLCM) was used to extract texture features and gray information from two-band ratio images K_782/999_. Finally, the MAM was proposed to detect the degree of bruising of loquats, which included the spectral data of three characteristic wavelength points in the NIR region coupled with texture features of the two-band ratio images, and the classification accuracy was 91.3%. This study shows that the MAM can be used as an effective dimensionality reduction method. The method not only improves the effect of prediction but also simplifies the process of prediction and ensures the accuracy of classification. The MSM can be used for rapid detection of normal and bruised fruits, and the MAM can be used to classify the degree of bruising of bruised fruits. Consequently, the processed methods are effective and can be used for the rapid and nondestructive detection of the degree of bruising of fruit.

## 1. Introduction

Bruising is one of the most important factors affecting the quality and price of fruit, and most consumers associate the absence of bruises on the surface of fruit with good quality. The bruised areas of fruit are more susceptible to decay, which infects normal fruits during storage and transportation [1], causing great economic losses. Due to enzyme or chemical oxidation of phenolic compounds, it takes several hours for bruised tissues to turn dark and brown [2], increasing transportation costs. Loquat was originally produced in China [3], is an economic fruit with both medicinal and food uses, and it has extremely high economic value [4]. Therefore, one of the main tasks of post-harvest processing of loquats is to detect whether loquats are bruised and the degree of bruising of loquats to reduce the loss by proper treatment. Selecting loquats based on their degree of bruising in advance can save storage and transportation costs. Lightly bruised loquats can be used to make loquat juice and loquat paste. If the loquat bruising is moderate, the damaged part will be removed, and the rest will be used to make canned loquat for preservation. Seriously bruised loquats are directly handled to save storage costs.

Currently, bruised loquats are often identified manually by the operators, who evaluate the surface bruise of fruit through naked eye observation and comparison with market quality standards [5]. The detection results of operators are affected by personal habits, light intensity, and subjective psychological factors, resulting in low efficiency, poor classification, and a high risk of human error in the sorting process. Therefore, they are not suitable for online fruit sorting in large quantities [6]. Hence, a new method is needed to detect the degree of bruising of loquats that has high precision, is fast, and is nondestructive.

In recent years, computer vision technology has been widely used in fruit surface detection [7], including mangoes [8], olives [9], oranges [10], and palm fruit [11]. These results indicate that the computer vision method combined with relevant algorithms can be used to effectively identify the surface damage in fruit. However, fruit is different in size, hardness, maturity, impact angle, and other parameters, resulting in different areas of bruised regions being generated when the same impact force is applied to the surface of fruit. For bruised fruit, there is only a slight change in the bruised area, and it cannot be classified by color characteristics. Therefore, it is difficult for the computer vision system to recognize lightly bruised fruit. The single color feature cannot effectively segment images, limiting the classification accuracy.

Hyperspectral imaging is an emerging technology, which combines traditional image and spectral technology. It is widely used in agricultural product detection [12]. Therefore, hyperspectral imaging technology may provide a means to identify and detect spectral and spatial anomalies of agricultural products. In previous studies, hyperspectral imaging has been widely used to identify specific damage in fruit, including decay lesions in citrus fruit [13], external insect infestations on jujube fruit [14], latent bruised damage in apples [15], early chilling injury in green bell peppers [16], and early decay in tomatoes [17]. Yuan et al. [18] used the interval variable iterative space contraction method (iVISSA) to reduce the dimensionality of the original spectral data; the PLS-DA model was established, and the detection accuracy of early bruises in jujubes was 100%. Sun et al. [19] used the successive projections algorithm (SPA) to reduce the dimension of the original spectral data, and the PLS-DA model was established to detect decayed honey peaches, with a classification accuracy of 98.75%. Cen et al. [20] used the sequential forward selection (SFS) method to reduce the dimensionality of the original spectral data; the SVM model was established to detect the chilling injury of cucumber, with a classification accuracy of 100%. Although hyperspectral imaging technology has a high detection accuracy, the measurement technology still needs a lot of time, so it is not suitable for online detection of agricultural fruit.

At present, principal component analysis (PCA) is used to reduce the dimensionality of hyperspectral data and to identify the several characteristic wavelength points that can be used for multispectral detection. Li et al. [21] presented the multispectral detection method, which was used to detect skin defects of ‘*Pinggu*’ peaches based on the Vis–NIR spectral region (400–1000 nm) hyperspectral data, and the PCA and two-band radio images were used to classify nine types of skin defects, with a correct classification rate of 96.6%. Zhang et al. [22] used PCA to reduce the dimensionality of the Vis–NIR region spectrum to obtain five characteristic wavelengths (540, 623, 675, 805, and 975 nm), which were used to detect defects in decayed citrus, with an accuracy of 97.73%. Xiong et al. [23] used PCA and GLCM to obtain the 7-dimension features of the PC3 image to classify different types of litchis, with a correct classification rate of 95%. The above study transformed hundreds of spectral data in the whole band of fruit into several wavelength points, or a specific PC image for analysis, which greatly saved detection time.

This study provides a multispectral analysis method for accurate identification of the degree of bruising of loquats. The purposes of this study are as follows:

(1) To explore the feasibility of detecting the degree of bruising of loquats by multispectral techniques in the hyperspectral NIR region.
(2) To confirm that the PC images in the hyperspectral NIR region can effectively distinguish bruised loquats from normal loquats.
(3) To determine if the spectral data of three characteristic wavelength points in the hyperspectral NIR region coupled with texture features and gray information of the two-band ratio images method can be used to effectively detect the degree of bruising of loquats.

## 2. Material and Methods

### 2.1. Loquat Samples

**Figure 1:**
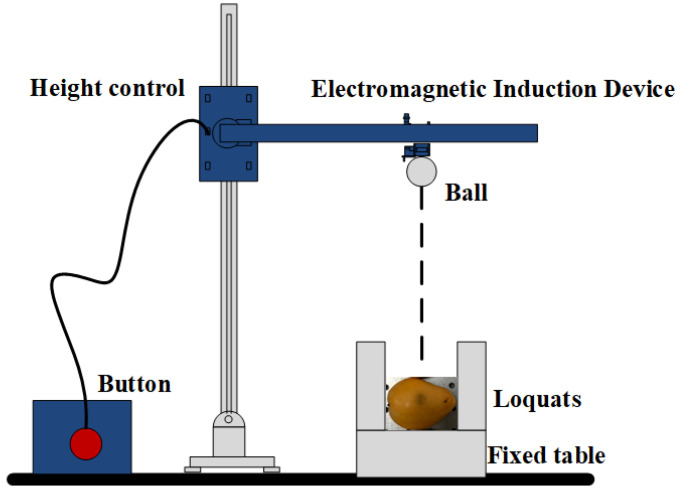
Free fall collision device.

**Figure 2:**
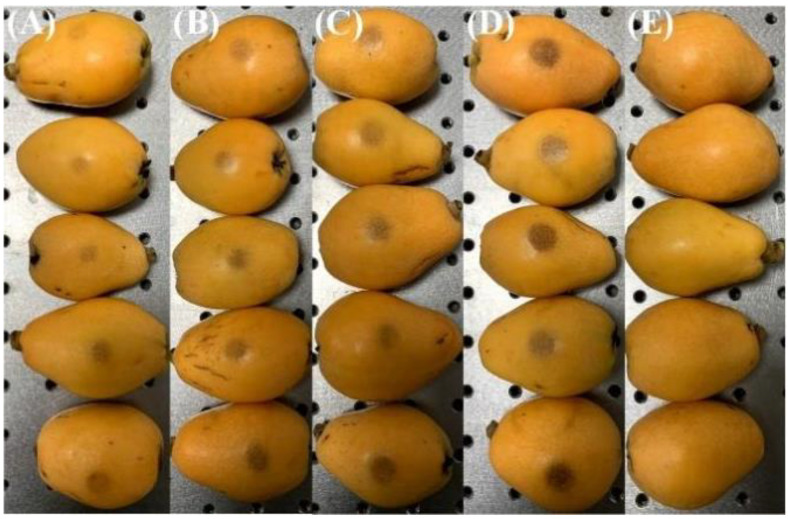
Loquat samples for (**A**) bruised grade I, (**B**) bruised grade II, (**C**) bruised grade III, (**D**) bruised grade IV, and (**E**) sound.

A total of 231 ‘*Zaozhong 6*’ loquats was purchased from a local ‘Shufeng’ orchard (Putian, China) in March 2022. To avoid the influence of other irrelevant factors, all the loquats selected for the experiment were similar in size (major axis diameter was about 80 mm and minor axis diameter was about 50 mm) and weight (about 70 g). Considering the collision of adjacent loquat areas to falls during harvesting, the fall height range of loquats was set at 0–40 cm. Adjusting the height to 0, 10, 20, 30, and 40 cm, five groups of samples were prepared by a ball hitting the equator of the loquat, as shown in Figure 1, which were labeled as sound (44), bruised grade I (50), bruised grade II (50), bruised grade III (47), and bruised grade IV (40) loquats, as shown in Figure 2. All samples were stored at room temperature of 24 ℃ for further processing.

### 2.2. Hyperspectral Image Acquisition System

**Figure 3:**
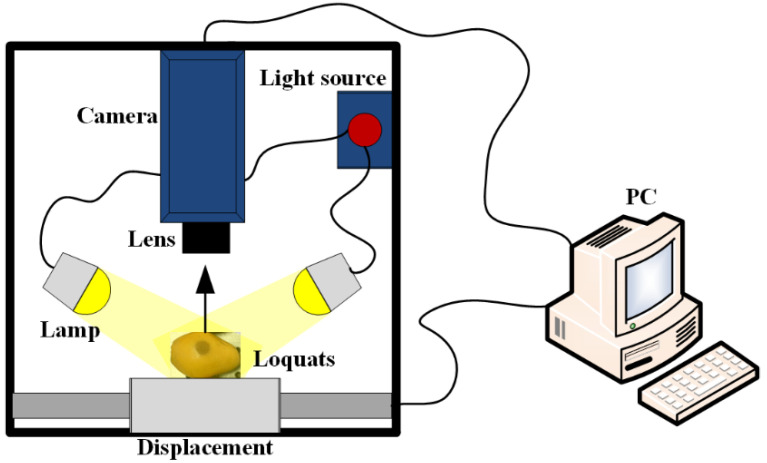
Hyperspectral imaging system.

As shown in Figure 3, the whole system (Gaia Sorter, Zolix, Beijing, China) consisted of an industrial camera (Hamamatsu C8484-05G, Hamamatsu, Japan), an imaging spectrograph (ImSpector V10E, Spectral Imaging Ltd., Oulu, Finland), an illumination system with four 20 W halogen area lamps (DECOSTAR51, MR16, OSRAM, Munich, Germany), an electrically controlled displacement platform, and a desk computer. The size of each of the acquired hyperspectral images was 960 × 488 pixels, and the bands were 176 at 3.4 nm intervals within the region of 397.5–1014 nm. Therefore, the acquired hyperspectral data were three-dimensional data (x,y,λ), where (x,y) is the pixel coordinates, and (λ) is the wavelength. Furthermore, the hyperspectral data were processed by ENVI 4.5 (Research System Inc., Boulder, CO, USA) and the image processing toolbox of MATLAB 2021a (The MathWorks Inc., Natick, MA, USA).

The hyperspectral system had to be preheated for 30 min before data collection to eliminate the influence of baseline drift and avoid errors in the image acquisition process. The parameters of the hyperspectral imaging system were adjusted by SpecView (Dualix Spectral Imaging, Wuxi, China), and the camera exposure time was set to 6 ms. The displacement stage advance speed of displacement stage advance was set to 1 cm·s^−1^. The displacement stage retreat speed was set to 2.5 cm·s^−1^ to save the sample acquisition time. Each loquat was scanned twice for data collection.

Due to the interference of dark current in the CCD camera, and the uneven distribution of light source intensity under each band, some bands with weaker light intensity contained more noise [24], so the acquired loquat hyperspectral images needed to be corrected by black and white reference image [25]. The relative reflectance was calculated by Formula (1):

(1) $$R_{xy}(\lambda )=\frac{T_{xy}(\lambda )-T_{d}(\lambda )}{T_{w}(\lambda )-T_{d}(\lambda )}$$

where *R_xy_*(*λ*), *T_xy_*(*λ*), *T_d_*(*λ*), and *T_w_*(*λ*) are the corrected hyperspectral image, the acquired original hyperspectral image, and the dark and white reference images, respectively.

### 2.3. Principal Component Analysis

Principal component analysis (PCA) is a multivariate statistical method that transforms the original high-dimensional data into linearly uncorrelated low-dimensional feature variables through an orthogonal transformation, and the transformed variables are called principal components (PCs). PCA is a linear algorithm; thus, it cannot be used to explain complex polynomial relationships between features. In general, the original data can be replaced by the first n PCs when the cumulative variance contribution of the current n PCs is sufficiently large.

In this paper, PCA was used to reduce the dimensionality of the corrected hyperspectral data of the loquats, and the several characteristic wavelengths discriminating the degree of bruising of loquat were selected by the weight coefficients. These principal component (PC) images were sorted according to the decreasing degree of contribution, and the first PC (PC1) scores image accounted for the most significant contribution rate.

The PCA was applied to perform the hyperspectral data of loquats in Vis (425–780 nm), NIR (781–1000 nm), and Vis–NIR (425–1000 nm), respectively. By visually comparing and analyzing the obtained PC images, the optimal spectral ranges for effectively segmenting the bruised and normal regions were determined. Before applying PCA, a binary mask generated by RGB images was created to produce an image containing only fruits, which was used to avoid interference from the background and improve recognition efficiency. Then, all specific PC images were visually evaluated to find the image that had the most significant contrast between the bruised area and the normal area.

### 2.4. Two-Band Radio Image

Each PC image was the linear sum of the original image of each wavelength multiplied by the corresponding weighting coefficients. Therefore, several local extreme points and wavelength points on both sides of the interval were selected as the characteristic wavelengths based on the weighting coefficients curve of the corresponding specific PC image. Subsequently, PCA was performed on the multispectral image composed of selected characteristic wavelength point images again, and the PC image with strong contrast was selected for further processing. In addition, several published studies have also shown that the band ratio images can be used to effectively enhance the contrast between bruised and normal areas, and a more uniform response on the surface of fruit can be produced by it [26]. Therefore, the band ratio image was used to develop the degree of bruising detection algorithm of bruised loquats in this study. The two-band ratio image is calculated by Formula (2):

(2) $$K_{a/b}=\frac{J_{a}}{J_{b}}$$

where *J_a_* and *J_b_* are the *a* and *b* wavelength original hyperspectral image, respectively, and *K_a/b_* is the two-band radio image.

### 2.5. Morphological Segmentation Method

**Figure 4:**
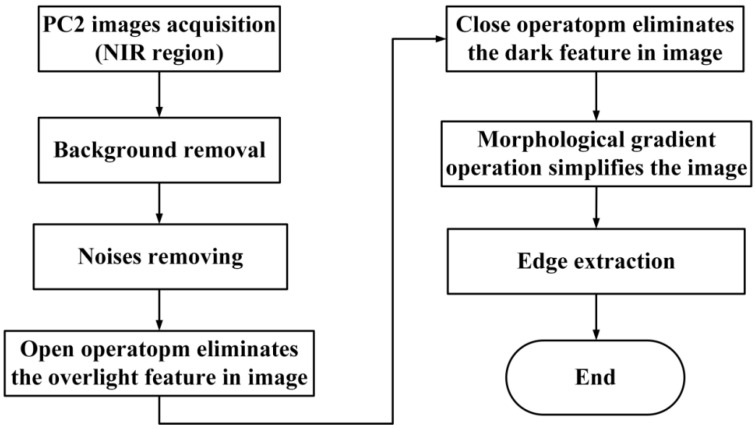
Flowchart of the MSM. MSM: morphological segmentation method. (PC2 image: the second PC scores image in NIR region).

The morphological segmentation method is proposed based on morphological processing, which includes morphological filtering and morphological gradient operation [27]. Erosion, dilation, opening, and closing operations are the basis of morphological filtering, and these operations can be used to remove weak noises, and reduce the effect of strong noises. The morphological opening operation can eliminate the light features. The closing operation can eliminate the dark features. Morphological gradient operation can enhance the contrast between a bruised area and a normal area, simplify the image boundary, and make the image smoother. However, the morphological gradient operation also enhances the noise in the image, which is eliminated by the morphological opening and closing operation, resulting in a simplified image with basic boundaries for further processing. A flowchart of the MSM is shown in Figure 4; the PC2 image of the NIR region was acquired after PCA was extracted, and the background information was removed by the binarization template of the original RGB image. Then, the ‘bwareaopen’ function was used to remove the noise in the image, and the opening and closing operations were used to eliminate the overly bright and dark feature points in the image. Finally, the morphology gradient operation and edge extraction algorithm were used to obtain the edge boundary of the sample.

### 2.6. Gray Level Co-Occurrence Matrix

Haralick et al. [28] proposed the statistical method of GLCM, which is a broad texture analysis method based on the premise that the spatial distribution relationship between pixels in an image contains the image texture information. Due to the large amount of data in GLCM, they are generally not directly used as texture features, but some statistics constructed based on them are used as texture classification features. In this study, the energy, entropy, contrast, correlation information, and inverse different moment features of each pixel point in four directions were extracted based on ‘Haralick’ features for subsequent processing [29].

### 2.7. Least Squares Support Vector Machine

The least squares support vector machine (LS-SVM) is a kernel function learning machine that follows the principle of structural risk minimization. It is an improvement of the SVM as the inequality constraint of SVM is replaced by an equation constraint. The sum of squared errors is used as the loss function, and the quadratic programming problem is transformed into a system of linear equations problem, which was used to improve the speed and convergence accuracy of the model.

### 2.8. Multispectral Analysis Method

**Figure 5:**
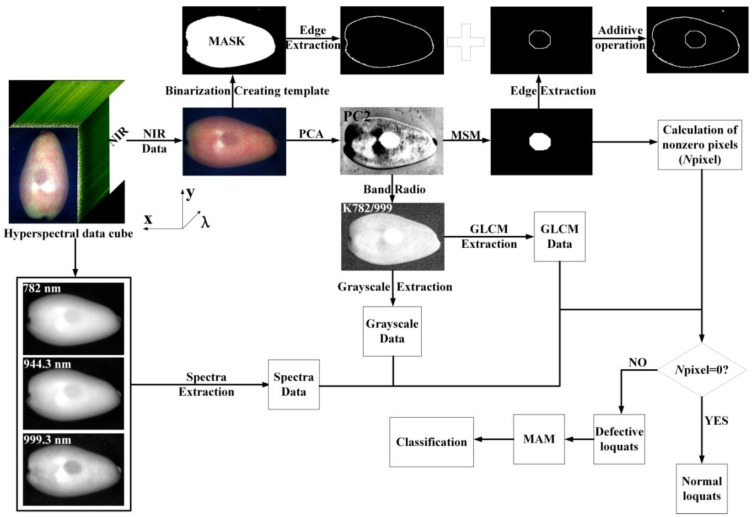
Flowchart of multispectral analysis method. (MSM: morphological segmentation method, MAM: multispectral analysis method, GLCM: gray level co-occurrence matrix).

The flowchart of the MAM based on the spectra of three characteristic wavelength points combined with the two-band radio image K_782/999_ is shown in Figure 5. Firstly, the original corrected hyperspectral in the NIR region was applied by PCA. Then, the MSM was used to segment the PC2 image with the most obvious bruised region, and only the segmentation results of the bruised area were retained. Furthermore, the number of non-zero pixels in the segmentation result was calculated. If it was zero, it was marked as a normal sample. If it was a non-zero value, it was marked as a bruised sample for subsequent processing. The weight curve of the PC2 image was analyzed, and the characteristic wavelength points (782 nm, 944.3 nm, 999.3 nm) were selected to obtain the two-band ratio images. Finally, the MAM based on the two-band ratio image K_782/999_ combined with the three characteristic wavelengths was used for subsequent classification of bruised loquats. As shown in Figure 5, the contrast between the bruised area and the normal area of the sample in the corresponding grayscale images of 782 nm, 944.3 nm, and 999.3 nm was far inferior to that of the PC2 image and band ratio image K_782/999_. At the same time, their illumination intensity had a heavy influence, which made the brightness of the images higher than the actual brightness, and the deviation to the segmentation of the bruised area in loquats was acquired. Although the PC2 image weakened the influence of light intensity, it still brought deviation. Therefore, the band ratio image was used to build a multispectral system in this study.

## 3. Results and Discussion

### 3.1. Spectra of ROIs in Samples

**Figure 6:**
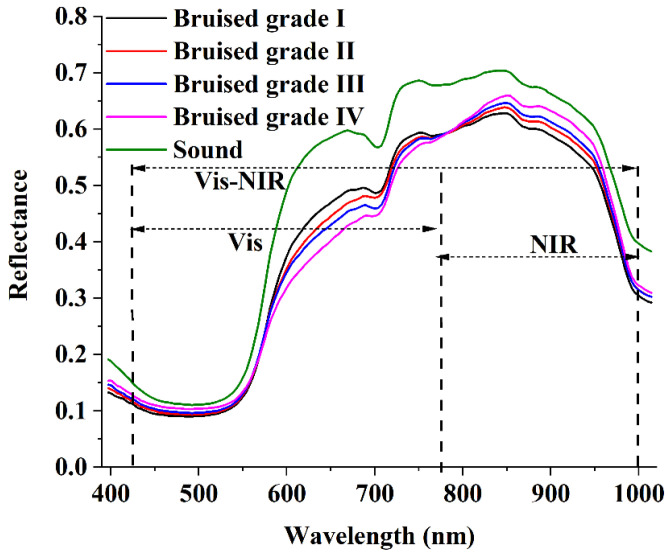
The average reflectance of loquats with different degrees of bruising.

Because every pixel in a hyperspectral image contains spectral information, the characteristic spectrum is extracted from a rectangular region of interest (ROI) containing about 100 pixels, which can reduce the error of spectral information difference between different pixels. The averages of reflectance of five degrees of bruising in the region of 397–1100 nm are shown in Figure 6, including bruised grade I, bruised grade II, bruised grade III, bruised grade IV, and sound loquat. As shown in Figure 5, the spectrum outside the range of 425–1000 nm had a significantly low SNR, which may have been caused by the dark current and low quantum efficiency of the CCD detector. Therefore, the images in the wavelength range of 425–1000 nm were used to subsequent analysis. All the spectra had similar characteristics and curve shapes, and the values of ROI reflectance were different for different degrees of bruising of loquats.

### 3.2. PCA in Vis–NIR Region

**Figure 7:**
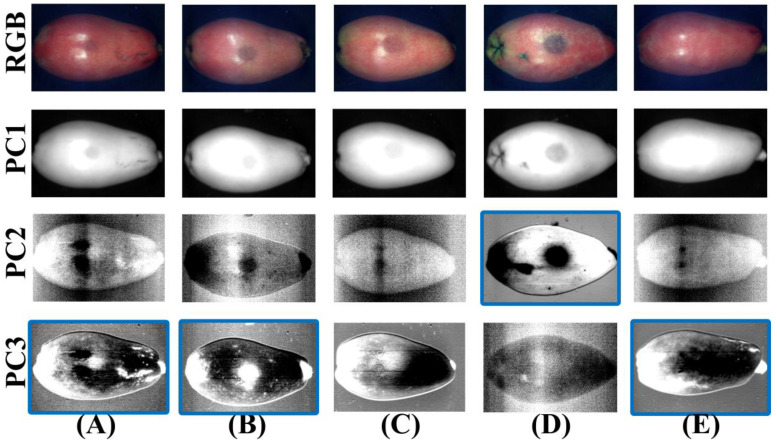
RGB and the PC images (PC1–PC3) in the Vis–NIR region for (**A**) bruised grade I, (**B**) bruised grade II, (**C**) bruised grade III, (**D**) bruised grade IV, and (**E**) sound. (Solid blue line: a clear contrast between the bruised and normal region in the PC image).

PCA is an effective means to reduce the dimensionality of hyperspectral data, and it can be used to enhance the contrast of ROIs and remove noise. PCA was performed on hyperspectral reflectance images of different degrees of bruising of samples in the Vis–NIR region, and the first three PC images were obtained, as shown in Figure 7. The subsequent PC images had too much noise, drowning the real information of loquats, and were not meaningful for bruised loquat detection, so they were not suitable for segmenting the bruised region in loquats. To make a more obvious contrast, the RGB images of loquats were placed in the first line and compared with RGB images. It could be seen in some PC images that the characteristics of the bruised region were more obvious. The PC1 image provided the average gray value information of the loquat over the entire spectral region and did not provide unique features of the bruised region in loquats. Therefore, the PC1 image is not suitable for detecting bruised loquats. However, it was also difficult to find a PC image that could be used to detect all degrees of bruising of loquats. Further studies showed a clear contrast between some bruised and normal regions in some specific PC images, which were manually marked using a solid blue line, as shown in Figure 7. Hence, these PC images could be used to distinguish specific degrees of bruising. The PC2 images could be used to detect bruised grade IV loquat. The PC3 images could be used to determine bruised grade I and II and sound loquats.

### 3.3. PCA in the Vis Region

**Figure 8:**
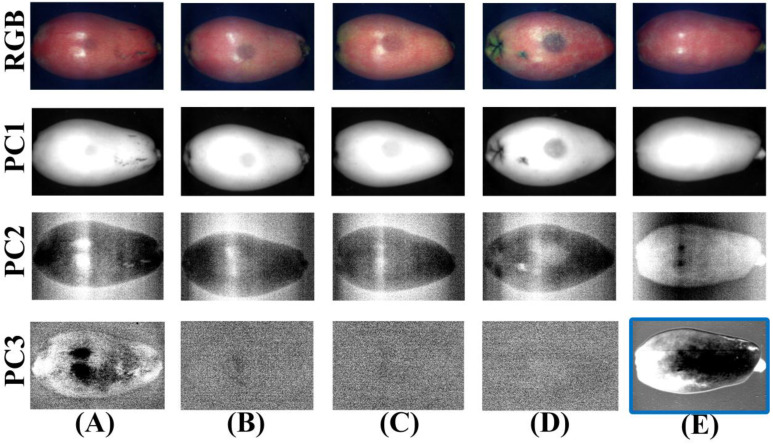
RGB and the PC images (PC1–PC3) in the Vis region for (**A**) bruised grade I, (**B**) bruised grade II, (**C**) bruised grade III, (**D**) bruised grade IV, and (**E**) sound. (Solid blue line: a clear contrast between the bruised and normal region in the PC image).

Generally, whether loquat is bruised or not can be directly judged by the naked eye, so it is easier to recognize in the Vis region, because the grayscale changes between bruised and normal regions are mainly affected by the spectra from this region. Therefore, the spectral data of the Vis region (Vis, 425–780 nm) was performed by PCA, and the generated PC images are shown in Figure 8. The first line of Figure 8 represents the RGB images of each bruised degree of loquats; the PC1 image could only reflect the grayscale information of the original samples. The sample information in PC2 and PC3 images was drowned by noise, and only sound loquats could be distinguished by the PC3 images. Therefore, the PC images in the Vis region cannot be used to effectively segment the bruised area of loquats based on visual evaluation and human eye identification.

### 3.4. PCA in the NIR Region

**Figure 9:**
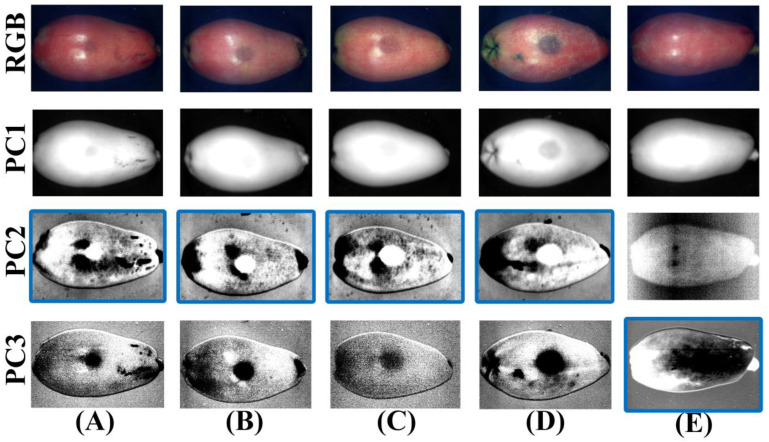
RGB and the PC images (PC1–PC3) in the NIR region for (**A**) bruised grade I, (**B**) bruised grade II, (**C**) bruised grade III, (**D**) bruised grade IV, and (**E**) sound. (Solid blue line: a clear contrast between the bruised and normal region in the PC image).

As shown in Figure 9, the results of the NIR region by PCA were better than the results of the Vis–NIR and Vis regions. The first line of Figure 9 shown the RGB images of different degrees of bruising of loquat; the PC1 images could only reflect the grayscale information of the original samples. The information in the PC3 image was drowned by noise, so they were not suitable for the segmentation of the bruised region of loquats. The PC2 images had the most obvious contrast in the bruised region of loquats, so they could be used to distinguish the degree of bruising of loquats. Although the PC2 images reflected the bruised information of loquats, the interference of light intensity information was brought by it, which needed to be eliminated in the subsequent processing. Gowen et al. [30] used PC2 images to detect bruised regions on mushroom surfaces; this study is consistent with Gowen’s conclusion.

### 3.5. Morphological Segmentation Method

**Figure 10:**
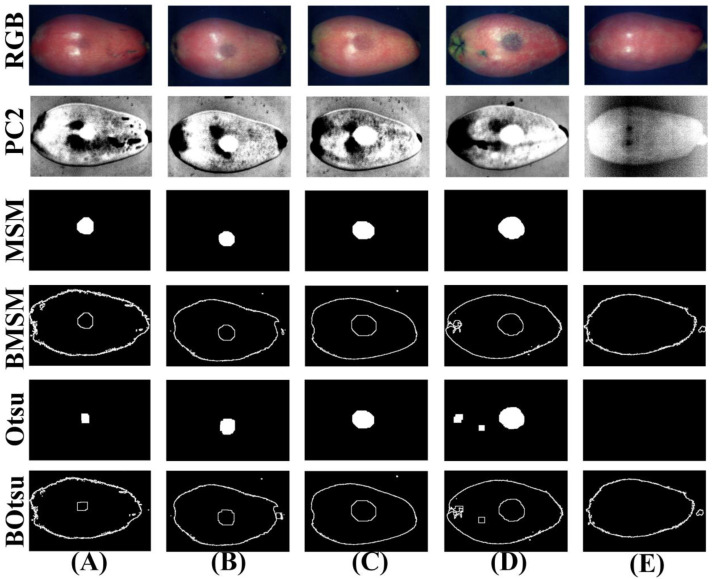
Segmentation results based on the PC2 images by using the MSM and Otsu for (**A**) bruised grade I, (**B**) bruised grade II, (**C**) bruised grade III, (**D**) bruised grade IV, and (**E**) sound. (MSM: morphological segmentation method, BMSM: boundary extraction based on MSM, BOtsu: boundary extraction based on Otsu).

To improve the detection efficiency of the multispectral system, the MSM was used to identify normal loquats, and the bruised region of loquats was used for subsequent processing. Firstly, the original corrected hyperspectral data in the NIR region was applied by PCA. Then, the MSM was used to segment the PC2 images with the most obvious bruised area of loquats, and only the segmentation results of bruised area were retained, as shown in Figure 10. Furthermore, the number of non-zero pixels in the segmentation result was calculated. If it was zero, it was marked as a normal sample. If it was a non-zero value, it was marked as a bruised sample for subsequent processing. As a comparison, the Otsu method was used to segment the PC2 images. The traditional Otsu method cannot completely segment the bruised region of fruit [31]. Compared with the results of the MSM proposed in this paper, the results of the Otsu are shown in Figure 10A. The Otsu method resulted in under-segmentation for bruised grade I and could only segment part of the bruised area of loquats. As shown in the Otsu in Figure 10D, the loquat with bruised grade IV was over-segmented, and the area outside the bruised area was segmented. The segmentation results for bruised grade II, bruised grade III, and normal loquats were good by the Otsu method. In fact, the loquats were different in size, hardness, maturity, impact angle, and other parameters, resulting in different areas of bruised regions being generated when the same impact force was applied to the surface of loquats. Therefore, it was difficult to classify different degrees of bruising of loquats by the number of pixels in the region. Hence, the MSM was used to discriminate between normal and bruised loquats, and it was not used for the degree of bruising of loquat classification.

### 3.6. PCA in the Multispectral Image

**Figure 11:**
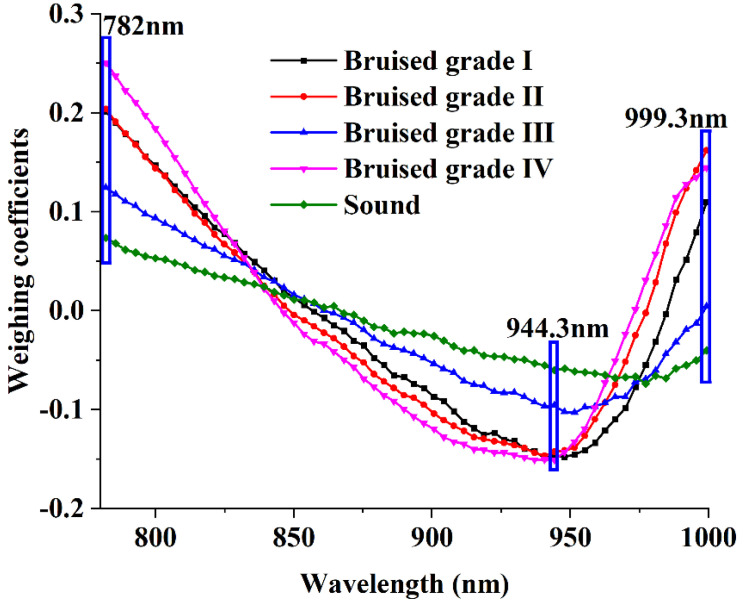
Weighting coefficients for PC2 images in the NIR region.

Weight coefficients at local peaks and valleys can be regarded as characteristic points, and the corresponding wavelength images were considered key wavelength images for classification [21]. In previous studies, the data of Vis and Vis–NIR regions could not classify the collision degree of fruit, so we only analyzed the data from the NIR region. Due to the individual differences of fruit, different fruit have different characteristic wavelengths [21,24]. The weight coefficients of different degrees of bruising of loquats are shown in Figure 11. There were clearly more difference at 782 nm, 944.3 nm, and 999.3 nm in the NIR region. The specific PC images corresponding to these important bands had obvious characteristics, so 782 nm, 944.3 nm, and 999.3 nm were selected as characteristic wavelengths (multispectral image) for further analysis.

**Figure 12:**
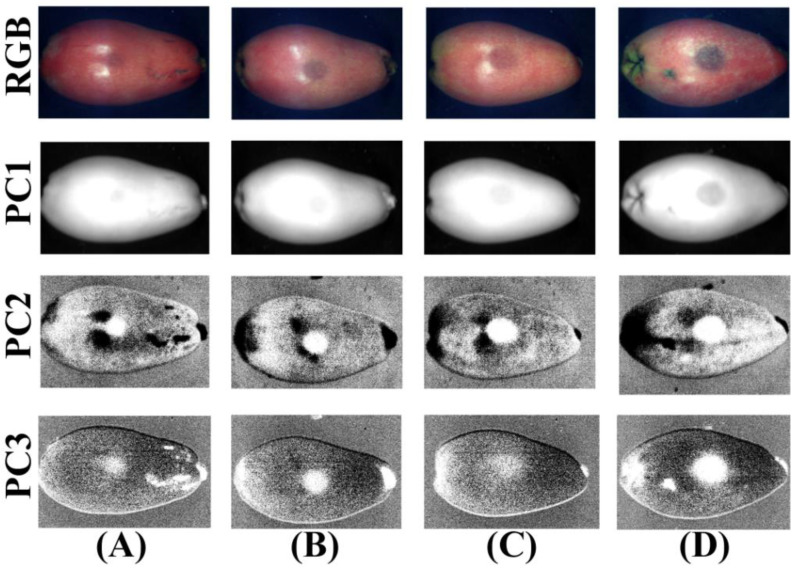
RGB and the PC images (PC1–PC3) in multispectral images from the NIR region for (**A**) bruised grade I, (**B**) bruised grade II, (**C**) bruised grade III, and (**D**) bruised grade IV.

In previous studies, the MSM was used to perfectly distinguish normal loquats from bruised loquats. In this study, we only discussed the bruised grade classification of bruised loquats. Based on the above analysis results, it was difficult to use the specific PC image by PCA in the Vis–NIR and Vis regions to detect all degrees of bruising of loquats. The PC2 images obtained by PCA in the NIR region could be used to detect all degrees of bruising of loquats. However, 65 wavelength points were still used for PCA in the NIR region, which took a lot of time in practical application. It was still a challenge to develop a multispectral system for the online detection of loquats. Therefore, the multispectral images of 782 nm, 944.3 nm, and 999.3 nm from the NIR region were used, and they were performed by PCA for further analysis; the results are shown in Figure 12.

Compared with the results in Figure 9, the contrast of the PC2 images of the bruised area of loquats was basically unchanged. However, the contrast of PC3 images between bruised and normal regions was better. The PC3 images could be used to accurately identify the root of loquats, and the influence of light was eliminated by it. However, it still had too much noise and could not be directly segmented, which requires further analysis.

### 3.7. Band Ratio Image

**Figure 13:**
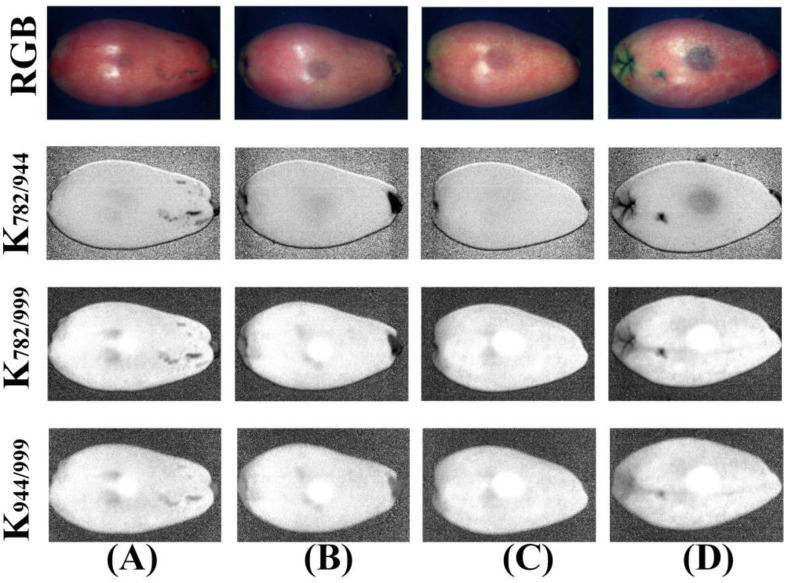
RGB and the two-band radio images (K_782/944_, K_782/999_, K_944/999_) in the multispectral image from the NIR region for (**A**) bruised grade I, (**B**) bruised grade II, (**C**) bruised grade III, and (**D**) bruised grade IV.

Further analysis of the NIR region weight curve was conducted, as shown in Figure 11. The weight coefficient values of the three characteristic wavelengths (782 nm, 944.3 nm, and 999.3 nm) had absolute differences. Therefore, better results may be obtained by analyzing the two-band ratio image [21,24]. The two-band ratio images (K_782/944_, K_782/999_, K_944/999_) with degree of bruising of loquats are shown in Figure 13. The contrasts of the two-band ratio images of K_782/944_ and K_782/999_ between the bruised areas and normal areas were clear. Compared with the PC images shown in Figure 10, the two-band ratio images had a more uniform response on the sample surface, eliminating the influence of light. However, the band ratio image K_944/999_ brought more noise in the background, and the contrast between bruised and normal regions was not as good as the band ratio image K_782/999_. Therefore, the two-band radio image K_782/999_ was the final target image. The multispectral analysis method was developed to classify four degree of bruising of loquats by using its average grayscale, texture features, and the spectra of three characteristic wavelength points (782 nm, 944.3 nm, and 999.3 nm).

### 3.8. Classification of Bruised Loquats

**Figure 14:**
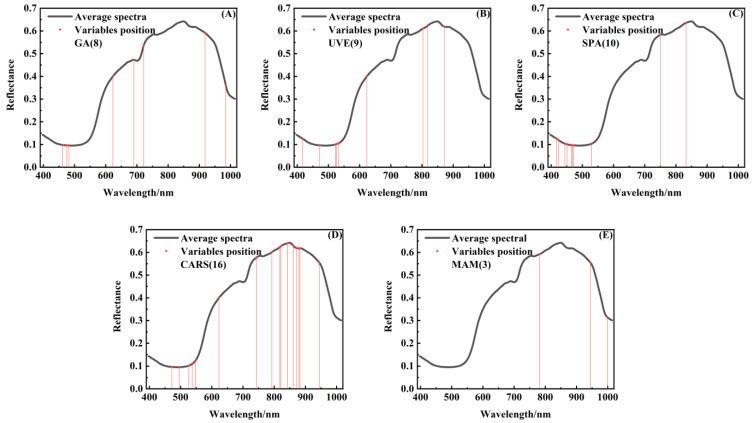
The locations of the reflectance spectra characteristic wavelengths were selected by (**A**) GA, (**B**) UVE, (**C**) SPA, (**D**) CARS, and (**E**) MAM. (MAM: multispectral analysis method).

In this study, the MAM was evaluated by 187 independent bruised loquats, and the LS-SVM algorithm was used to verify the performance of various band selection methods. The results of classification are shown in Table 1. Four degrees of bruising of loquats with bruised grades I (50), II (50), III (47), and IV (40) were studied. The samples were divided into modeling and prediction sets by the Kennard–Stone method with a ratio of 3:1 [32]. In this study, different band selection methods were used to reduce the dimensionality of the original spectral data to improve the detection speed. The number of spectra by genetic algorithm (GA) [33], uninformative variable elimination (UVE) [34], successive projections algorithm (SPA) [35], competitive adaptive reweighted sampling (CARS) [36], and the multispectral analysis method (MAM) were 8, 9, 10, 16, and 3, respectively, as shown in Figure 14. Compared with the number of characteristic spectra selected by these scholars [33,34,35,36], the MAM proposed in this paper used the least number of spectra.

Based on the differences of kernel function, and band selection methods, the LS-SVM models were established based on spectra (S-LS-SVM), and spectra combined with texture features (S-T-LS-SVM). The kernel functions included the radial basis function (RBF), and linear kernel function (Lin) [37]. As shown in Table 1, the overall accuracy of the LS-SVM model based on RBF (RBF-LS-SVM) was better than the LS-SVM model based on the LIN kernel function (LIN-LS-SVM). The accuracy of the S-T-LS-SVM model was better than the S-LS-SVM model for all band selection methods.

In the RBF-S-T-LS-SVM model, the classification accuracy of Raw, GA, UVE, SPA, CARS, and MAM processed data were 91.30%, 86.96%, 86.96%, 89.13%, 89.13%, and 91.30%, respectively. Although the Raw-RBF-S-T-LS-SVM model had the same accuracy as MAM-RBF-S-T-LS-SVM, only three spectral features were used in the MAM, which greatly saved detection time. Therefore, the performance of the MAM model was better. Li et al. [38] used the LS-SVM algorithm to classify the bruising time of peaches. Although his research showed that the accuracy rate for bruised 36 h of peaches was 100%, it used 176 spectra, which would consume a lot of time in practical application. The above results indicate that the MAM proposed in this study can achieve the highest accuracy with the minimum number of spectra. It provides a possibility for rapid online detection of agricultural products.

**Figure 15:**
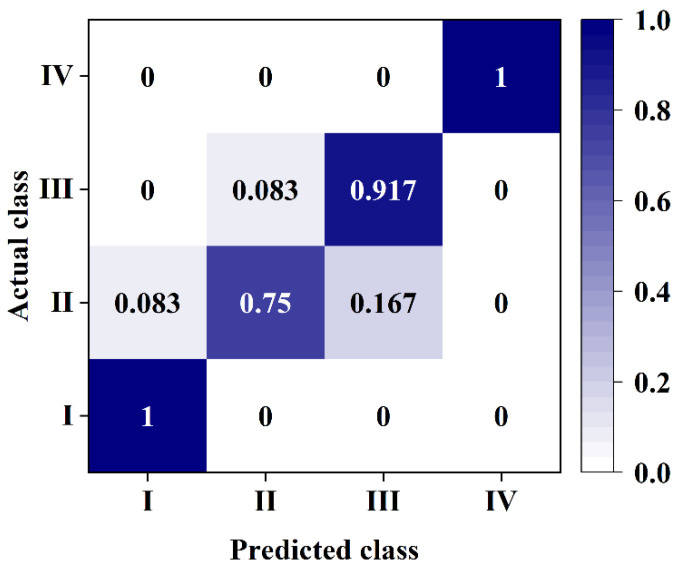
Classification results of the MAM-RBF-LS-SVM model.

Further analysis of the classification results of the MAM-RBF-LS-SVM model showed that the bruised grade I and IV loquats could be 100% recognized. The misjudged samples mainly came from the bruised grade II loquats and the bruised grade III loquats, as shown in Figure 15. It can be seen from Figure 15 that in the group of bruised grade II of loquats, 75% of the samples were identified, 8.3% of the samples were misclassified as bruised grade I, and 16.7% of the samples were misclassified as bruised grade III.

In the group of bruised grade III loquats, 91.7% of the samples were identified, and 8.3% of the samples were misclassified as bruised grade II. Observing and analyzing the misjudgment samples, we found that the loquats were different in hardness, shape, and maturity, so the damage of the bruised grade II samples was close to that of the bruised grade I or III samples, resulting in misjudgment. Based on the above analysis, the classification model can be optimized by improving the discriminant accuracy of the bruised grade II group.

## 4. Conclusions

In this study, the hyperspectral data of Vis–NIR (425–1000 nm), Vis (425–780 nm), and NIR (781–1000 nm) regions were used to evaluate the classification performance of the bruised and normal loquats, and the NIR region was the best. The MSM was proposed to classify the bruised loquat and normal loquat, which could improve the speed of detection and facilitate the transportation of normal loquats. Finally, the MAM was proposed to effectively discriminate four degrees of bruising of loquats, with an overall accuracy of 91.30%. These results indicate that the proposed methodology based on hyperspectral imaging is a promising tool to assess the quality of loquat fruits. The MSM can be used for the rapid detection of normal and bruised fruits, and the MAM can be used to classify the degree of bruising of bruised fruits, significantly reducing the online detection time. The new methods proposed in this study can be used to realize the rapid, non-destructive, and high precision online detection of fruit.

## Figures and Tables

**Table 1:** Classification results of characteristic wavelengths based on the LS-SVM model.

Method	Number	Accuracy (%)			
		RBF		LIN	
		S-LS-SVM	S-T-LS-SVM	S-LS-SVM	S-T-LS-SVM
Raw	172	89.13	**91.30**	89.13	89.13
GA	8	86.96	**86.96**	80.43	80.43
UVE	9	82.61	**86.96**	80.43	80.43
SPA	10	82.61	**89.13**	80.43	82.61
CARS	16	84.78	**89.13**	84.78	84.78
MAM	3	**91.30**		80.43	

RBF: radial basis function; LIN: LIN kernel function; S-LS-SVM: LS-SVM model based on spectra; S-T-LS-SVM: LS-SVM model based on spectra combining texture features.

## Data Availability

The data presented in this study are available on request from the corresponding author.
